# Naming ang Comprehension Features in Language of Schizophrenia Adolescents. Nouns and Verbs Task

**DOI:** 10.1192/j.eurpsy.2022.908

**Published:** 2022-09-01

**Authors:** E. Shvedovskiy, N. Zvereva, E. Balakireva

**Affiliations:** 1 Mental Health Research Center, Medical Psychology, Moscow, Russian Federation; 2 Mental Health Research Center, Child Psychiatry, Moscow, Russian Federation

**Keywords:** Naming, comprehension, schizophrénia, Adolescents

## Abstract

**Introduction:**

Naming and comprehension are standing amog the basic language functions, which allow individuals to realize the communication domain of language. Naming and comprehension imparements are well-studied (Sebastian et al., 2018) in most affected patient groups (for example aphasia patients), but at the same time schizophrenia process may cause it’s specific language disorders (Andreasen et al., 1985). Adolescent age is a very sensitive period in the context of beginning of schizophrenia.

**Objectives:**

The purpose of present study was to identify which language imparement (naming or comprehension) is the most affected in adolescent non-psychotic schizophrenia in this age. Also, authors were aimed at the check of selected tool sensitivness to schizophrenia patients

**Methods:**

Subjects of present study were patients with schizophrenia of Moscow psychiatry clinic (n=20, mean age=14,4), subdivided by DS (F20.xx, F21.xx) and syndromes (national Russian psychiatric subdivision inside the DS). All DS and syndromes were additionally qualified by the clinical professional. Following methods were used: medical history analysis (expert diagnosisqualification, syndromic analysis), Test “Quantative Language Assessment in Aphasia” (QLAA) (Tsvetkova et al., 1981), statistical analysis. QLAA consists from the 4 subtests: naming of objects (NO), actions (NA); comprehension of objects (CO), actions (CA). Answers were quantified by the 3-mark scale (0-0,5-1).

**Results:**

Mean QLAA NO = 14,5; NA = 14,5; CO = 16; CA = 19. Ingroup comparison using U-criteria showed that differences between NO and CA are the most significant (p<0.05). Differences in all other pairs are not so significant.

**Conclusions:**

language comprehension is studied group of adolescent patients with schizophrenia is the most affected language domain

**Disclosure:**

No significant relationships.

